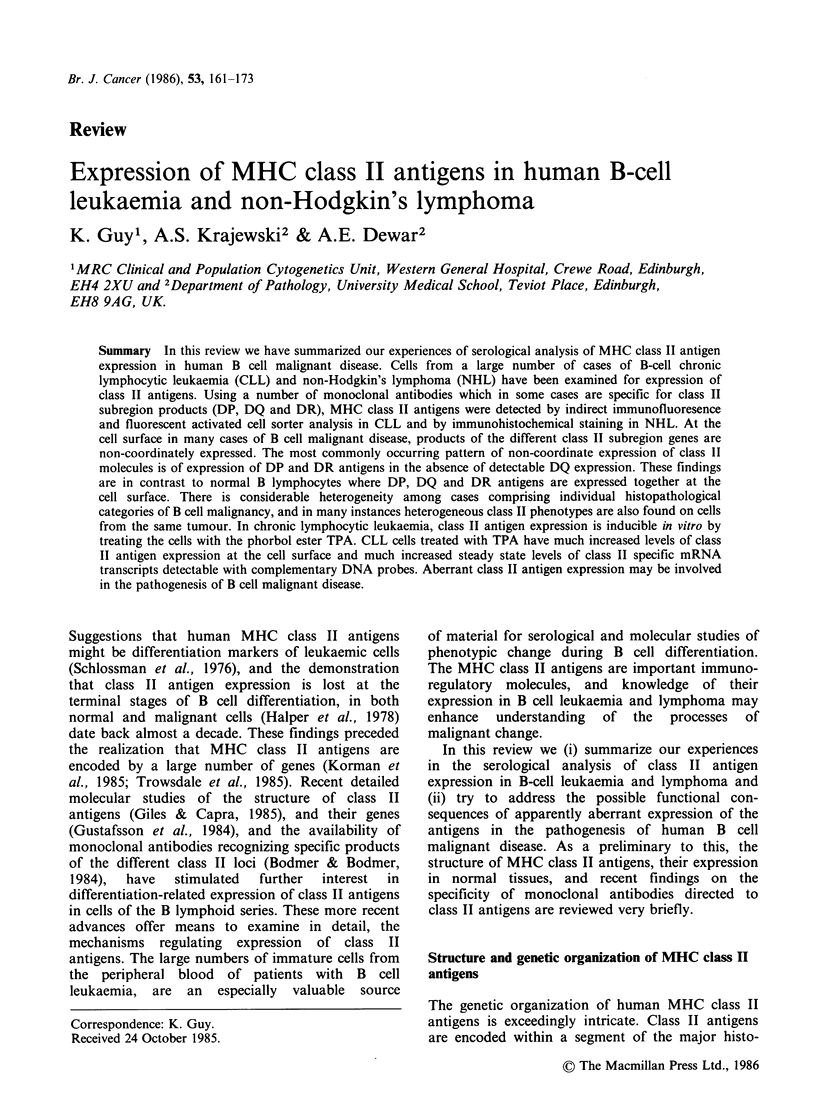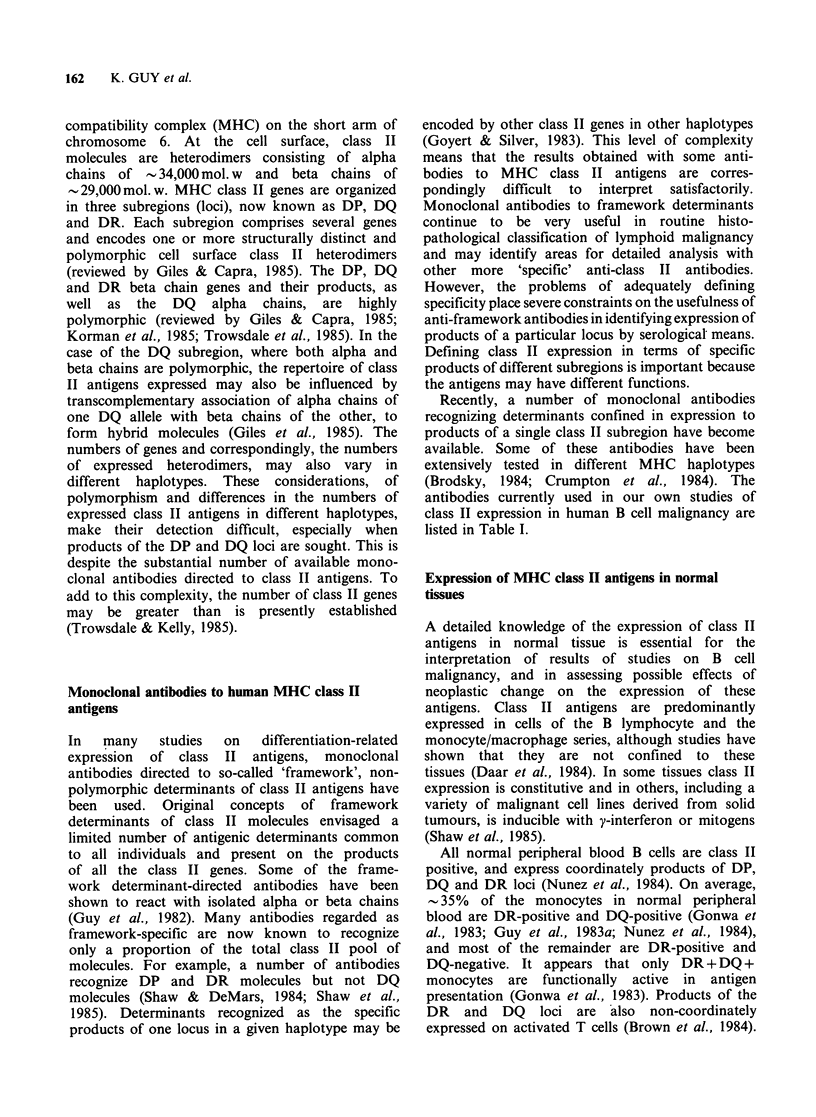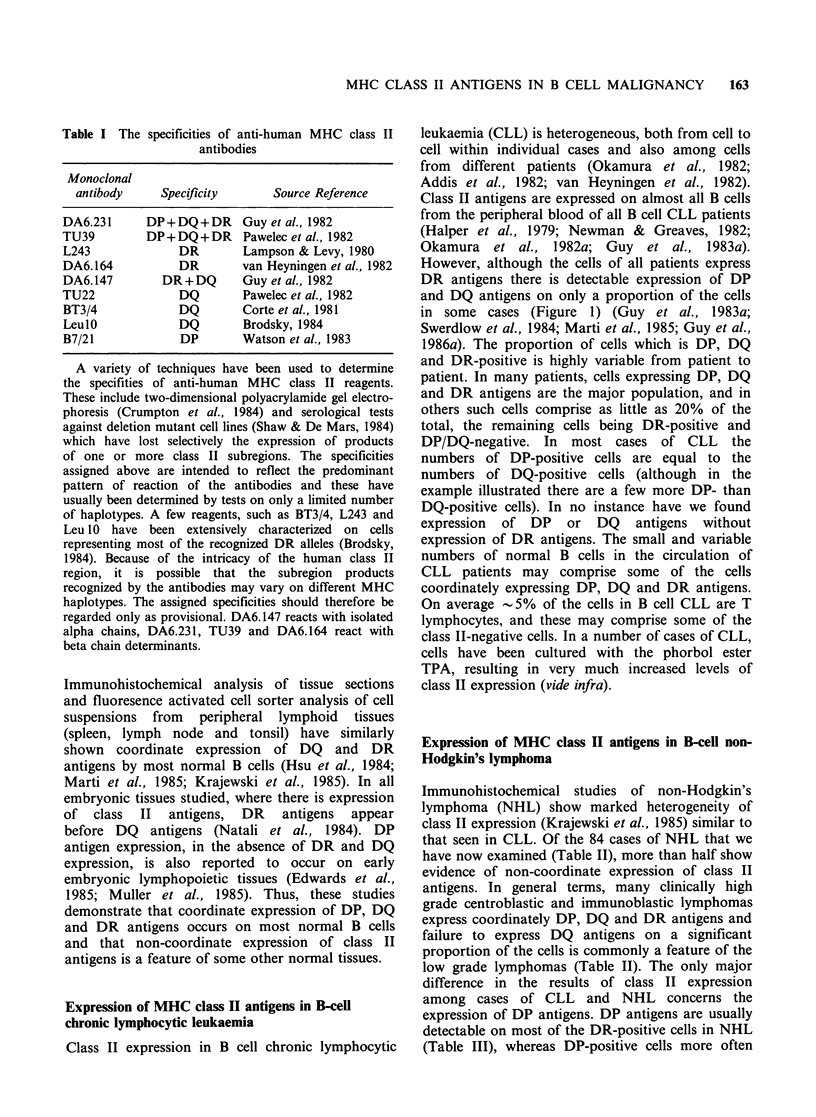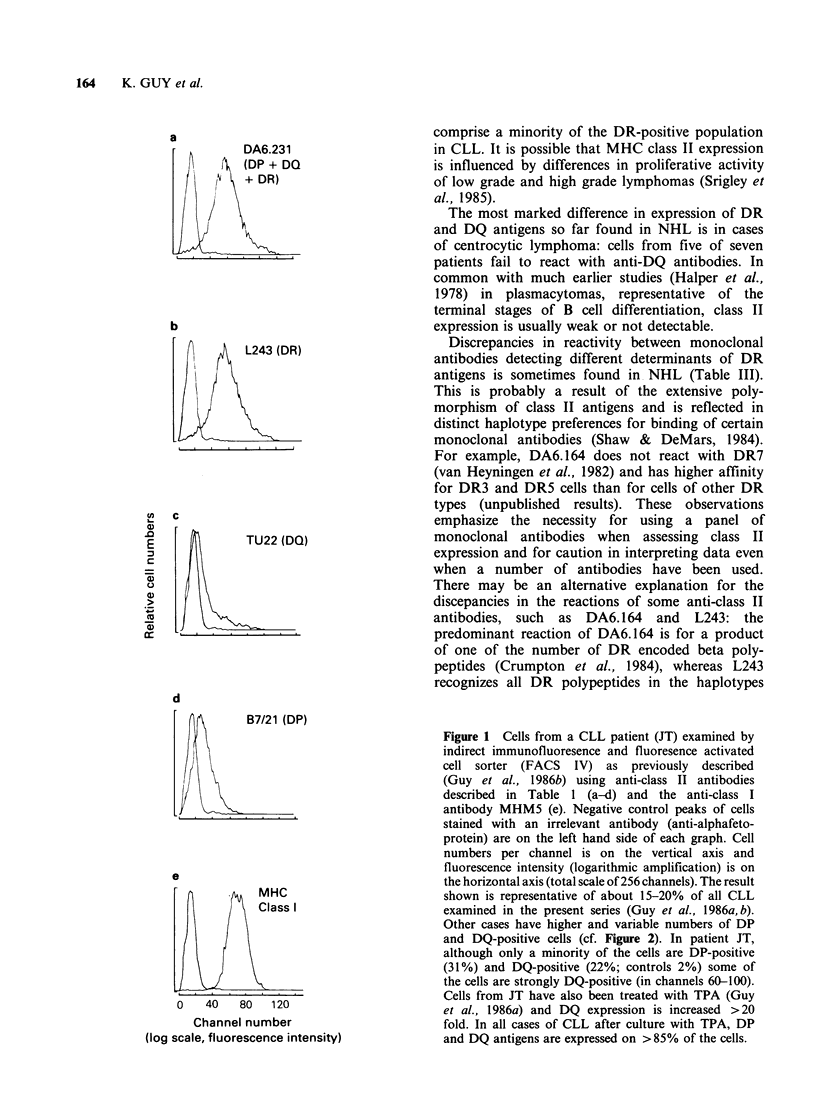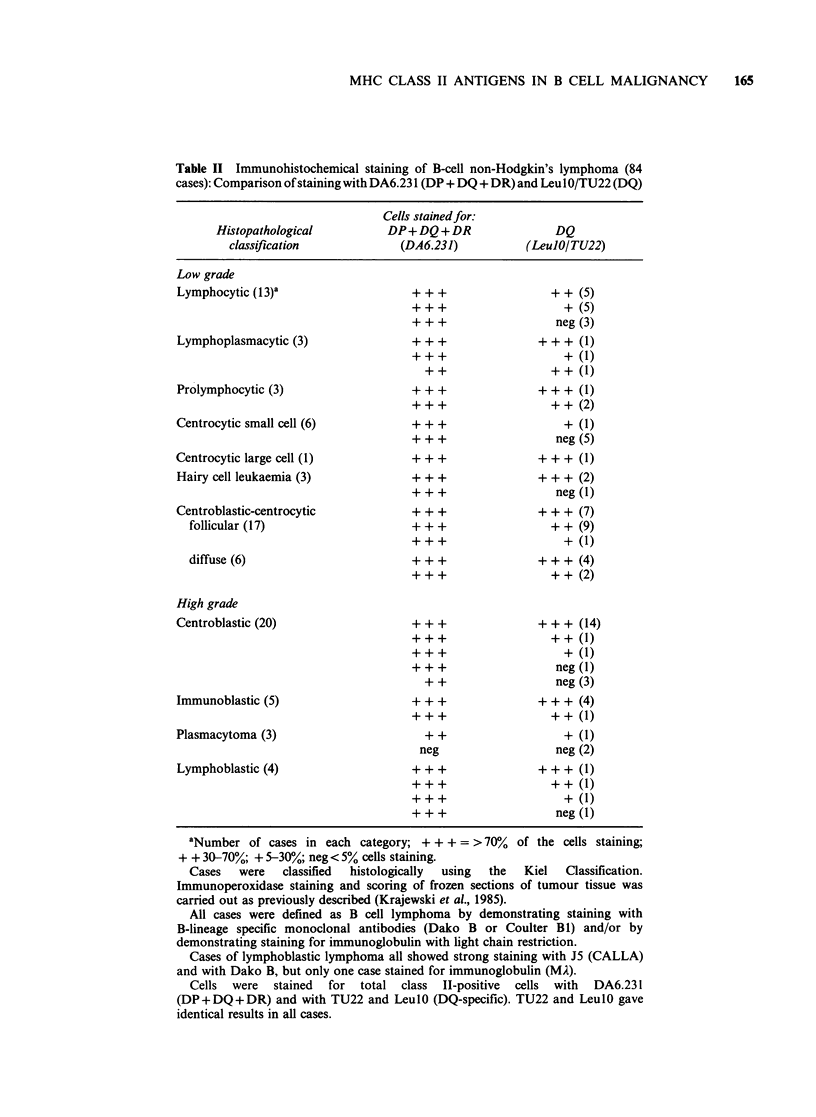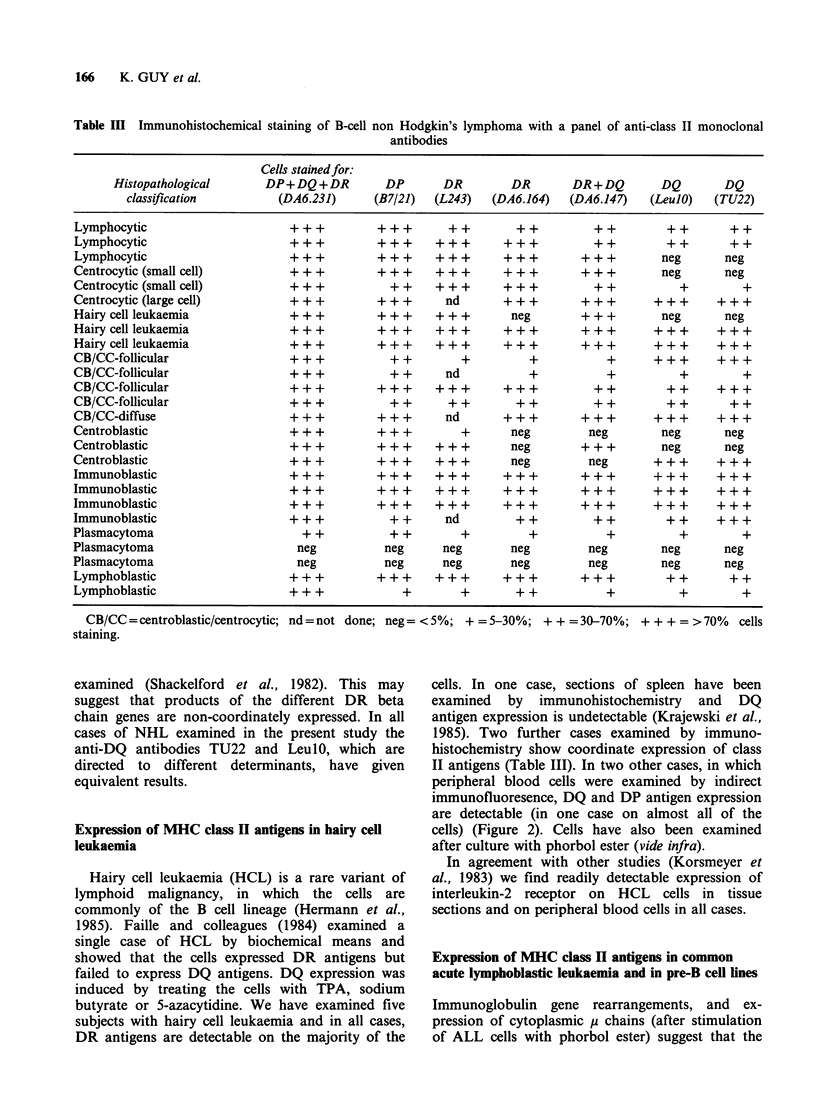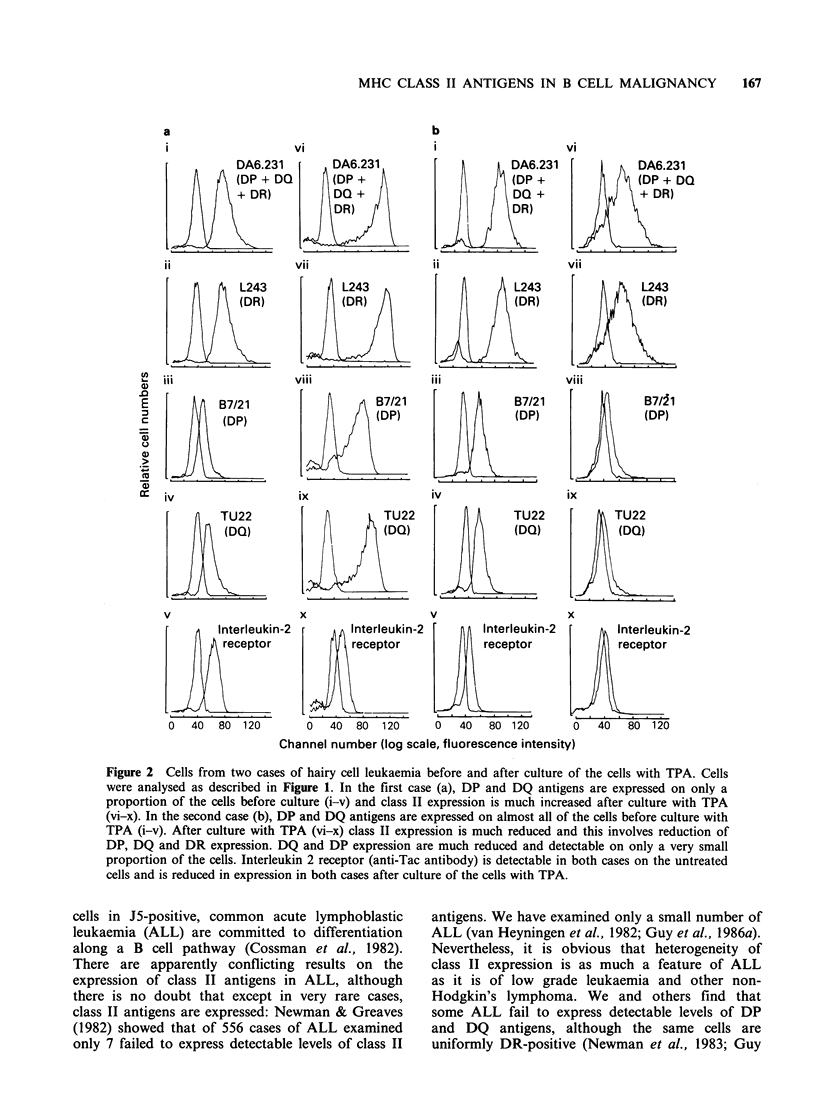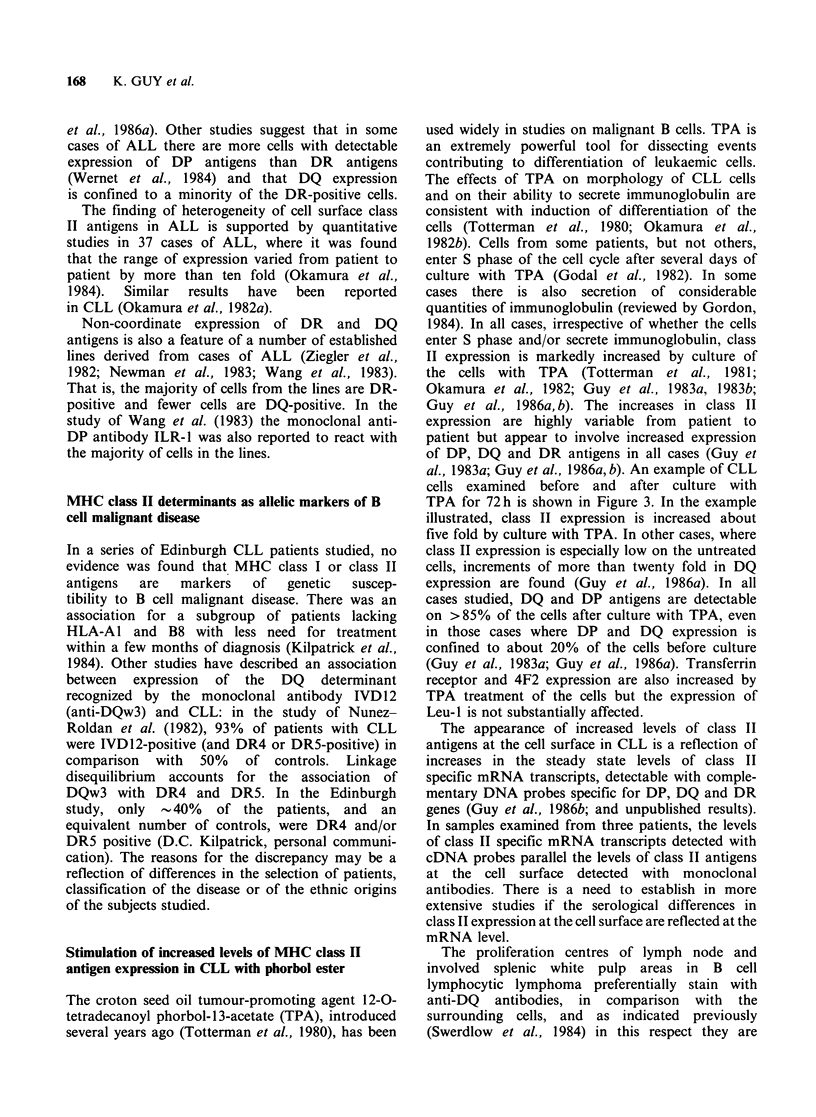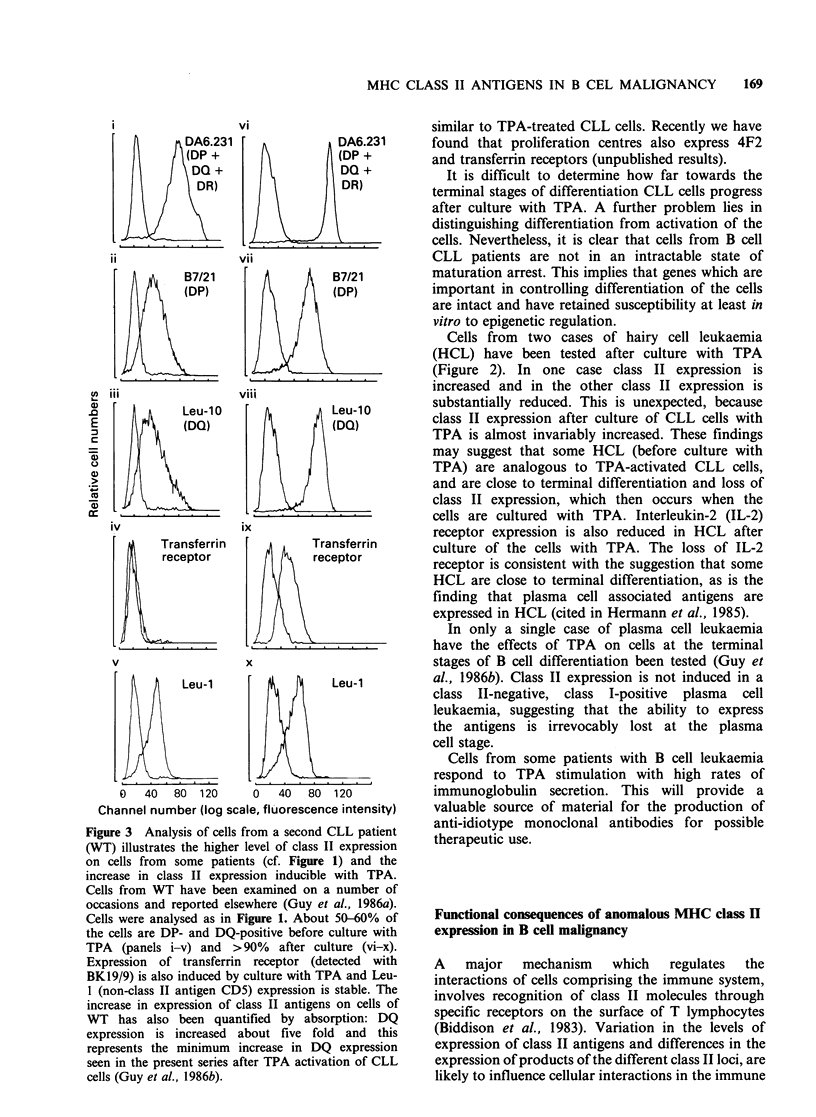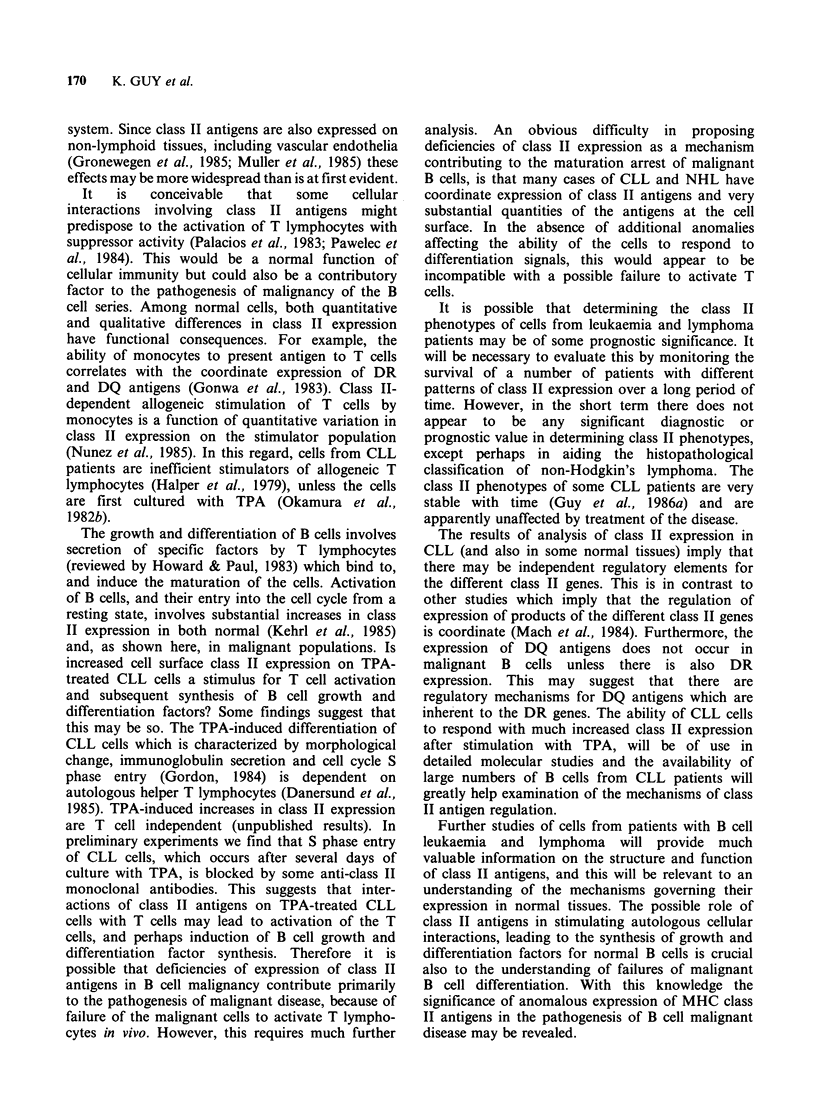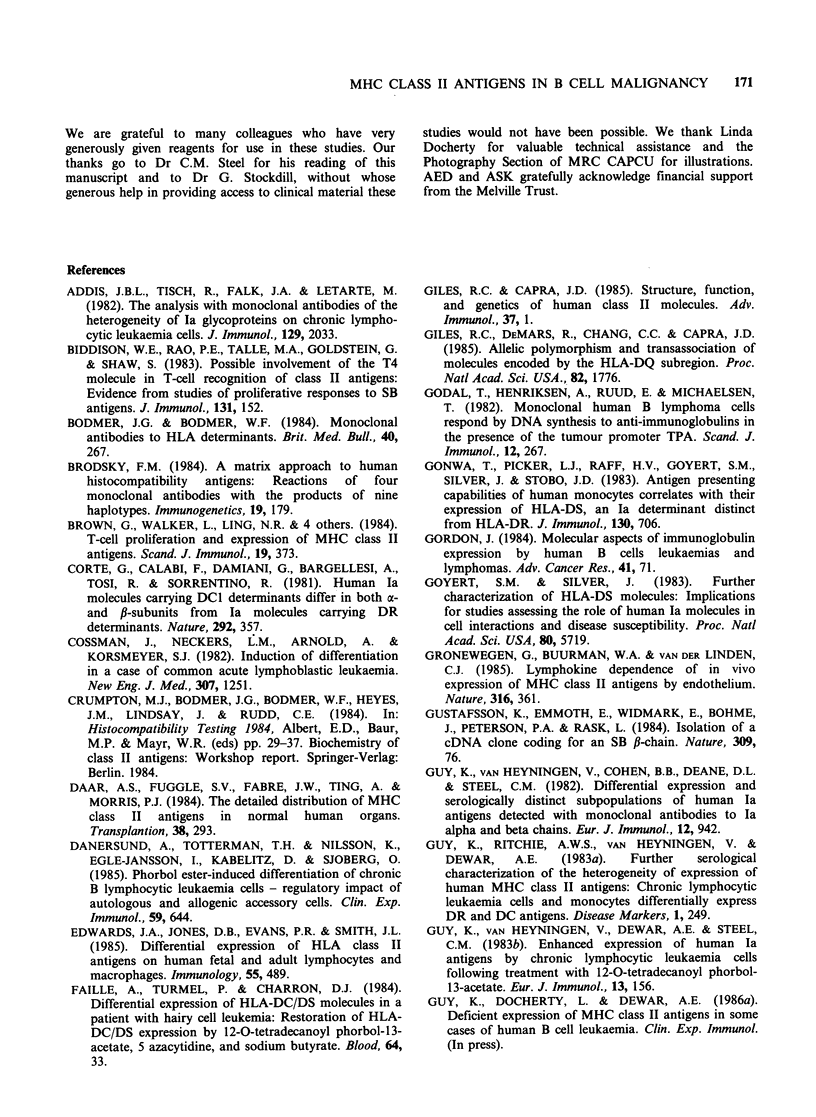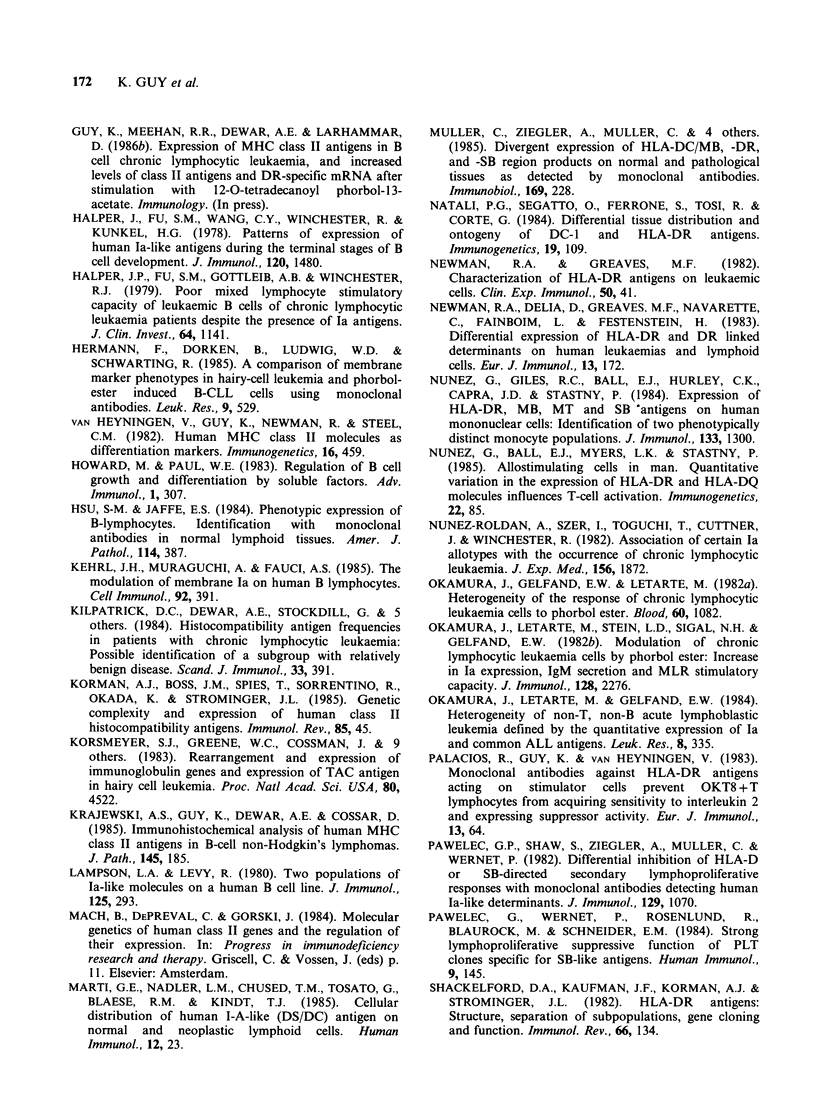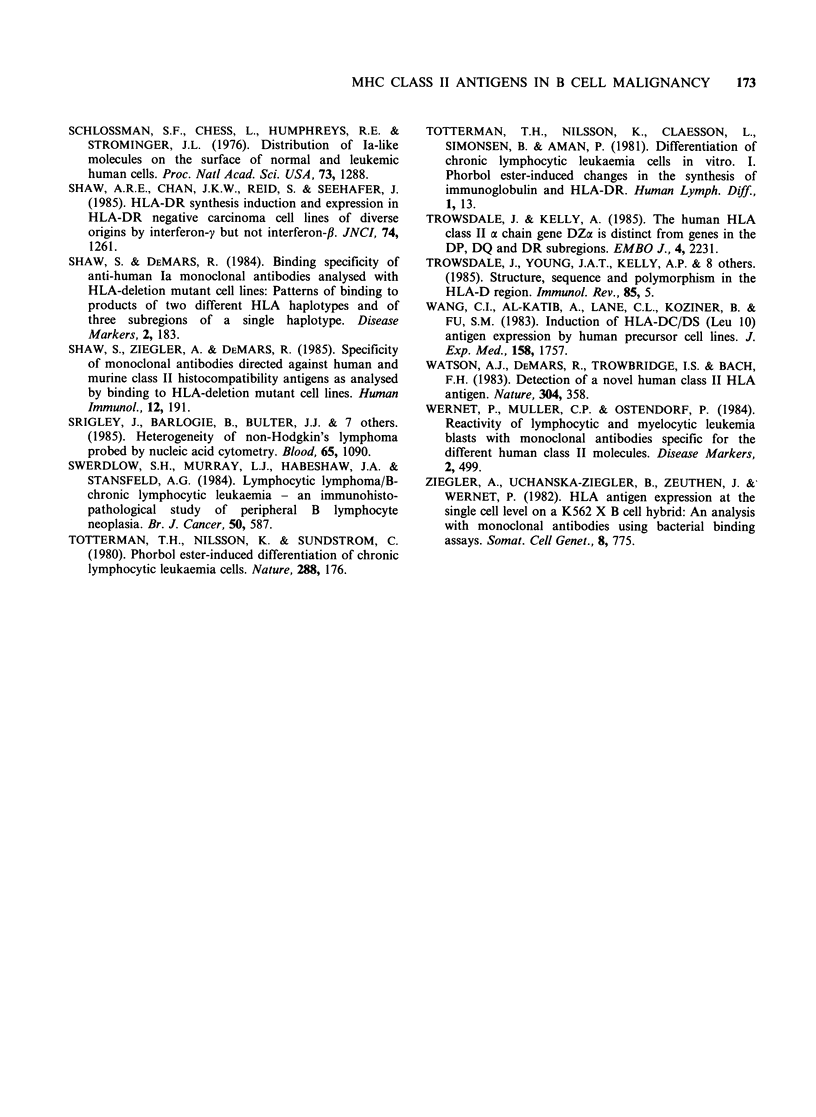# Expression of MHC class II antigens in human B-cell leukaemia and non-Hodgkin's lymphoma.

**DOI:** 10.1038/bjc.1986.31

**Published:** 1986-02

**Authors:** K. Guy, A. S. Krajewski, A. E. Dewar

## Abstract

In this review we have summarized our experiences of serological analysis of MHC class II antigen expression in human B cell malignant disease. Cells from a large number of cases of B-cell chronic lymphocytic leukaemia (CLL) and non-Hodgkin's lymphoma (NHL) have been examined for expression of class II antigens. Using a number of monoclonal antibodies which in some cases are specific for class II subregion products (DP, DQ and DR), MHC class II antigens were detected by indirect immunofluorescence and fluorescent activated cell sorter analysis in CLL and by immunohistochemical staining in NHL. At the cell surface in many cases of B cell malignant disease, products of the different class II subregion genes are non-coordinately expressed. The most commonly occurring pattern of non-coordinate expression of class II molecules is of expression of DP and DR antigens in the absence of detectable DQ expression. These findings are in contrast to normal B lymphocytes where DP, DQ and DR antigens are expressed together at the cell surface. There is considerable heterogeneity among cases comprising individual histopathological categories of B cell malignancy, and in many instances heterogeneous class II phenotypes are also found on cells from the same tumour. In chronic lymphocytic leukaemia, class II antigen expression is inducible in vitro by treating the cells with the phorbol ester TPA. CLL cells treated with TPA have much increased levels of class II antigen expression at the cell surface and much increased steady state levels of class II specific mRNA transcripts detectable with complementary DNA probes. Aberrant class II antigen expression may be involved in the pathogenesis of B cell malignant disease.


*Br. J. Cancer* (1986), **53**, 161–173

**Review**

### Expression of MHC class II antigens in human B-cell leukaemia and non-Hodgkin's lymphoma

K. Guy[1], A.S. Krajewski[2] & A.E. Dewar[2]

[1]*MRC Clinical and Population Cytogenetics Unit, Western General Hospital, Crewe Road, Edinburgh, EH4 2XU and* [2]*Department of Pathology, University Medical School, Teviot Place, Edinburgh, EH8 9AG, UK.*

**Summary** In this review we have summarized our experiences of serological analysis of MHC class II antigen expression in human B cell malignant disease. Cells from a large number of cases of B-cell chronic lymphocytic leukaemia (CLL) and non-Hodgkin's lymphoma (NHL) have been examined for expression of class II antigens. Using a number of monoclonal antibodies which in some cases are specific for class II subregion products (DP, DQ and DR), MHC class II antigens were detected by indirect immunofluoresence and fluorescent activated cell sorter analysis in CLL and by immunohistochemical staining in NHL. At the cell surface in many cases of B cell malignant disease, products of the different class II subregion genes are non-coordinately expressed. The most commonly occurring pattern of non-coordinate expression of class II molecules is of expression of DP and DR antigens in the absence of detectable DQ expression. These findings are in contrast to normal B lymphocytes where DP, DQ and DR antigens are expressed together at the cell surface. There is considerable heterogeneity among cases comprising individual histopathological categories of B cell malignancy, and in many instances heterogeneous class II phenotypes are also found on cells from the same tumour. In chronic lymphocytic leukaemia, class II antigen expression is inducible *in vitro* by treating the cells with the phorbol ester TPA. CLL cells treated with TPA have much increased levels of class II antigen expression at the cell surface and much increased steady state levels of class II specific mRNA transcripts detectable with complementary DNA probes. Aberrant class II antigen expression may be involved in the pathogenesis of B cell malignant disease.

Suggestions that human MHC class II antigens might be differentiation markers of leukaemic cells (Schlossman *et al.*, 1976), and the demonstration that class II antigen expression is lost at the terminal stages of B cell differentiation, in both normal and malignant cells (Halper *et al.*, 1978) date back almost a decade. These findings preceded the realization that MHC class II antigens are encoded by a large number of genes (Korman *et al.*, 1985; Trowsdale *et al.*, 1985). Recent detailed molecular studies of the structure of class II antigens (Giles & Capra, 1985), and their genes (Gustafsson *et al.*, 1984), and the availability of monoclonal antibodies recognizing specific products of the different class II loci (Bodmer & Bodmer, 1984), have stimulated further interest in differentiation-related expression of class II antigens in cells of the B lymphoid series. These more recent advances offer means to examine in detail, the mechanisms regulating expression of class II antigens. The large numbers of immature cells from the peripheral blood of patients with B cell leukaemia, are an especially valuable source of material for serological and molecular studies of phenotypic change during B cell differentiation. The MHC class II antigens are important immuno-regulatory molecules, and knowledge of their expression in B cell leukaemia and lymphoma may enhance understanding of the processes of malignant change.

In this review we (i) summarize our experiences in the serological analysis of class II antigen expression in B-cell leukaemia and lymphoma and (ii) try to address the possible functional consequences of apparently aberrant expression of the antigens in the pathogenesis of human B cell malignant disease. As a preliminary to this, the structure of MHC class II antigens, their expression in normal tissues, and recent findings on the specificity of monoclonal antibodies directed to class II antigens are reviewed very briefly.

### Structure and genetic organization of MHC class II antigens

The genetic organization of human MHC class II antigens is exceedingly intricate. Class II antigens are encoded within a segment of the major histo-

Correspondence: K. Guy.
Received 24 October 1985.

© The Macmillan Press Ltd., 1986

compatibility complex (MHC) on the short arm of chromosome 6. At the cell surface, class II molecules are heterodimers consisting of alpha chains of ~34,000 mol. w and beta chains of ~29,000 mol. w. MHC class II genes are organized in three subregions (loci), now known as DP, DQ and DR. Each subregion comprises several genes and encodes one or more structurally distinct and polymorphic cell surface class II heterodimers (reviewed by Giles & Capra, 1985). The DP, DQ and DR beta chain genes and their products, as well as the DQ alpha chains, are highly polymorphic (reviewed by Giles & Capra, 1985; Korman *et al.*, 1985; Trowsdale *et al.*, 1985). In the case of the DQ subregion, where both alpha and beta chains are polymorphic, the repertoire of class II antigens expressed may also be influenced by transcomplementary association of alpha chains of one DQ allele with beta chains of the other, to form hybrid molecules (Giles *et al.*, 1985). The numbers of genes and correspondingly, the numbers of expressed heterodimers, may also vary in different haplotypes. These considerations, of polymorphism and differences in the numbers of expressed class II antigens in different haplotypes, make their detection difficult, especially when products of the DP and DQ loci are sought. This is despite the substantial number of available monoclonal antibodies directed to class II antigens. To add to this complexity, the number of class II genes may be greater than is presently established (Trowsdale & Kelly, 1985).

### Monoclonal antibodies to human MHC class II antigens

In many studies on differentiation-related expression of class II antigens, monoclonal antibodies directed to so-called 'framework', non-polymorphic determinants of class II antigens have been used. Original concepts of framework determinants of class II molecules envisaged a limited number of antigenic determinants common to all individuals and present on the products of all the class II genes. Some of the framework determinant-directed antibodies have been shown to react with isolated alpha or beta chains (Guy *et al.*, 1982). Many antibodies regarded as framework-specific are now known to recognize only a proportion of the total class II pool of molecules. For example, a number of antibodies recognize DP and DR molecules but not DQ molecules (Shaw & DeMars, 1984; Shaw *et al.*, 1985). Determinants recognized as the specific products of one locus in a given haplotype may be encoded by other class II genes in other haplotypes (Goyert & Silver, 1983). This level of complexity means that the results obtained with some antibodies to MHC class II antigens are correspondingly difficult to interpret satisfactorily. Monoclonal antibodies to framework determinants continue to be very useful in routine histopathological classification of lymphoid malignancy and may identify areas for detailed analysis with other more 'specific' anti-class II antibodies. However, the problems of adequately defining specificity place severe constraints on the usefulness of anti-framework antibodies in identifying expression of products of a particular locus by serological means. Defining class II expression in terms of specific products of different subregions is important because the antigens may have different functions.

Recently, a number of monoclonal antibodies recognizing determinants confined in expression to products of a single class II subregion have become available. Some of these antibodies have been extensively tested in different MHC haplotypes (Brodsky, 1984; Crumpton *et al.*, 1984). The antibodies currently used in our own studies of class II expression in human B cell malignancy are listed in Table I.

### Expression of MHC class II antigens in normal tissues

A detailed knowledge of the expression of class II antigens in normal tissue is essential for the interpretation of results of studies on B cell malignancy, and in assessing possible effects of neoplastic change on the expression of these antigens. Class II antigens are predominantly expressed in cells of the B lymphocyte and the monocyte/macrophage series, although studies have shown that they are not confined to these tissues (Daar *et al.*, 1984). In some tissues class II expression is constitutive and in others, including a variety of malignant cell lines derived from solid tumours, is inducible with $\gamma$-interferon or mitogens (Shaw *et al.*, 1985).

All normal peripheral blood B cells are class II positive, and express coordinately products of DP, DQ and DR loci (Nunez *et al.*, 1984). On average, ~35% of the monocytes in normal peripheral blood are DR-positive and DQ-positive (Gonwa *et al.*, 1983; Guy *et al.*, 1983*a*; Nunez *et al.*, 1984), and most of the remainder are DR-positive and DQ-negative. It appears that only DR+DQ+ monocytes are functionally active in antigen presentation (Gonwa *et al.*, 1983). Products of the DR and DQ loci are also non-coordinately expressed on activated T cells (Brown *et al.*, 1984).

**Table I** The specificities of anti-human MHC class II antibodies

| *Monoclonal antibody* | *Specificity* | *Source Reference* |
|---|---|---|
| DA6.231 | DP+DQ+DR | Guy *et al.*, 1982 |
| TU39 | DP+DQ+DR | Pawelec *et al.*, 1982 |
| L243 | DR | Lampson & Levy, 1980 |
| DA6.164 | DR | van Heyningen *et al.*, 1982 |
| DA6.147 | DR+DQ | Guy *et al.*, 1982 |
| TU22 | DQ | Pawelec *et al.*, 1982 |
| BT3/4 | DQ | Corte *et al.*, 1981 |
| Leu10 | DQ | Brodsky, 1984 |
| B7/21 | DP | Watson *et al.*, 1983 |

A variety of techniques have been used to determine the specifities of anti-human MHC class II reagents. These include two-dimensional polyacrylamide gel electrophoresis (Crumpton *et al.*, 1984) and serological tests against deletion mutant cell lines (Shaw & De Mars, 1984) which have lost selectively the expression of products of one or more class II subregions. The specificities assigned above are intended to reflect the predominant pattern of reaction of the antibodies and these have usually been determined by tests on only a limited number of haplotypes. A few reagents, such as BT3/4, L243 and Leu 10 have been extensively characterized on cells representing most of the recognized DR alleles (Brodsky, 1984). Because of the intricacy of the human class II region, it is possible that the subregion products recognized by the antibodies may vary on different MHC haplotypes. The assigned specificities should therefore be regarded only as provisional. DA6.147 reacts with isolated alpha chains, DA6.231, TU39 and DA6.164 react with beta chain determinants.

Immunohistochemical analysis of tissue sections and fluoresence activated cell sorter analysis of cell suspensions from peripheral lymphoid tissues (spleen, lymph node and tonsil) have similarly shown coordinate expression of DQ and DR antigens by most normal B cells (Hsu *et al.*, 1984; Marti *et al.*, 1985; Krajewski *et al.*, 1985). In all embryonic tissues studied, where there is expression of class II antigens, DR antigens appear before DQ antigens (Natali *et al.*, 1984). DP antigen expression, in the absence of DR and DQ expression, is also reported to occur on early embryonic lymphopoietic tissues (Edwards *et al.*, 1985; Muller *et al.*, 1985). Thus, these studies demonstrate that coordinate expression of DP, DQ and DR antigens occurs on most normal B cells and that non-coordinate expression of class II antigens is a feature of some other normal tissues.

### Expression of MHC class II antigens in B-cell chronic lymphocytic leukaemia

Class II expression in B cell chronic lymphocytic leukaemia (CLL) is heterogeneous, both from cell to cell within individual cases and also among cells from different patients (Okamura *et al.*, 1982; Addis *et al.*, 1982; van Heyningen *et al.*, 1982). Class II antigens are expressed on almost all B cells from the peripheral blood of all B cell CLL patients (Halper *et al.*, 1979; Newman & Greaves, 1982; Okamura *et al.*, 1982*a*; Guy *et al.*, 1983*a*). However, although the cells of all patients express DR antigens there is detectable expression of DP and DQ antigens on only a proportion of the cells in some cases (Figure 1) (Guy *et al.*, 1983*a*; Swerdlow *et al.*, 1984; Marti *et al.*, 1985; Guy *et al.*, 1986*a*). The proportion of cells which is DP, DQ and DR-positive is highly variable from patient to patient. In many patients, cells expressing DP, DQ and DR antigens are the major population, and in others such cells comprise as little as 20% of the total, the remaining cells being DR-positive and DP/DQ-negative. In most cases of CLL the numbers of DP-positive cells are equal to the numbers of DQ-positive cells (although in the example illustrated there are a few more DP- than DQ-positive cells). In no instance have we found expression of DP or DQ antigens without expression of DR antigens. The small and variable numbers of normal B cells in the circulation of CLL patients may comprise some of the cells coordinately expressing DP, DQ and DR antigens. On average $\sim$5% of the cells in B cell CLL are T lymphocytes, and these may comprise some of the class II-negative cells. In a number of cases of CLL, cells have been cultured with the phorbol ester TPA, resulting in very much increased levels of class II expression (*vide infra*).

### Expression of MHC class II antigens in B-cell non-Hodgkin's lymphoma

Immunohistochemical studies of non-Hodgkin's lymphoma (NHL) show marked heterogeneity of class II expression (Krajewski *et al.*, 1985) similar to that seen in CLL. Of the 84 cases of NHL that we have now examined (Table II), more than half show evidence of non-coordinate expression of class II antigens. In general terms, many clinically high grade centroblastic and immunoblastic lymphomas express coordinately DP, DQ and DR antigens and failure to express DQ antigens on a significant proportion of the cells is commonly a feature of the low grade lymphomas (Table II). The only major difference in the results of class II expression among cases of CLL and NHL concerns the expression of DP antigens. DP antigens are usually detectable on most of the DR-positive cells in NHL (Table III), whereas DP-positive cells more often comprise a minority of the DR-positive population in CLL. It is possible that MHC class II expression is influenced by differences in proliferative activity of low grade and high grade lymphomas (Srigley *et al.*, 1985).

The most marked difference in expression of DR and DQ antigens so far found in NHL is in cases of centrocytic lymphoma: cells from five of seven patients fail to react with anti-DQ antibodies. In common with much earlier studies (Halper *et al.*, 1978) in plasmacytomas, representative of the terminal stages of B cell differentiation, class II expression is usually weak or not detectable.

Discrepancies in reactivity between monoclonal antibodies detecting different determinants of DR antigens is sometimes found in NHL (Table III). This is probably a result of the extensive polymorphism of class II antigens and is reflected in distinct haplotype preferences for binding of certain monoclonal antibodies (Shaw & DeMars, 1984). For example, DA6.164 does not react with DR7 (van Heyningen *et al.*, 1982) and has higher affinity for DR3 and DR5 cells than for cells of other DR types (unpublished results). These observations emphasize the necessity for using a panel of monoclonal antibodies when assessing class II expression and for caution in interpreting data even when a number of antibodies have been used. There may be an alternative explanation for the discepancies in the reactions of some anti-class II antibodies, such as DA6.164 and L243: the predominant reaction of DA6.164 is for a product of one of the number of DR encoded beta polypeptides (Crumpton *et al.*, 1984), whereas L243 recognizes all DR polypeptides in the haplotypes

**Figure 1** Cells from a CLL patient (JT) examined by indirect immunofluoresence and fluoresence activated cell sorter (FACS IV) as previously described (Guy *et al.*, 1986*b*) using anti-class II antibodies described in Table 1 (a–d) and the anti-class I antibody MHM5 (e). Negative control peaks of cells stained with an irrelevant antibody (anti-alphafetoprotein) are on the left hand side of each graph. Cell numbers per channel is on the vertical axis and fluorescence intensity (logarithmic amplification) is on the horizontal axis (total scale of 256 channels). The result shown is representative of about 15–20% of all CLL examined in the present series (Guy *et al.*, 1986*a,b*). Other cases have higher and variable numbers of DP and DQ-positive cells (cf. **Figure 2**). In patient JT, although only a minority of the cells are DP-positive (31%) and DQ-positive (22%; controls 2%) some of the cells are strongly DQ-positive (in channels 60–100). Cells from JT have also been treated with TPA (Guy *et al.*, 1986*a*) and DQ expression is increased >20 fold. In all cases of CLL after culture with TPA, DP and DQ antigens are expressed on >85% of the cells.

**Table II** Immunohistochemical staining of B-cell non-Hodgkin's lymphoma (84 cases): Comparison of staining with DA6.231 (DP + DQ + DR) and Leu10/TU22 (DQ)

| *Histopathological classification* | *Cells stained for: DP+DQ+DR (DA6.231)* | *DQ (Leu10/TU22)* |
|---|---|---|
| *Low grade* | | |
| Lymphocytic (13)[a] | +++ | ++ (5) |
| | +++ | + (5) |
| | +++ | neg (3) |
| Lymphoplasmacytic (3) | +++ | +++ (1) |
| | +++ | + (1) |
| | ++ | ++ (1) |
| Prolymphocytic (3) | +++ | +++ (1) |
| | +++ | ++ (2) |
| Centrocytic small cell (6) | +++ | + (1) |
| | +++ | neg (5) |
| Centrocytic large cell (1) | +++ | +++ (1) |
| Hairy cell leukaemia (3) | +++ | +++ (2) |
| | +++ | neg (1) |
| Centroblastic-centrocytic follicular (17) | +++ | +++ (7) |
| | +++ | ++ (9) |
| | +++ | + (1) |
| diffuse (6) | +++ | +++ (4) |
| | +++ | ++ (2) |
| *High grade* | | |
| Centroblastic (20) | +++ | +++ (14) |
| | +++ | ++ (1) |
| | +++ | + (1) |
| | +++ | neg (1) |
| | ++ | neg (3) |
| Immunoblastic (5) | +++ | +++ (4) |
| | +++ | ++ (1) |
| Plasmacytoma (3) | ++ | + (1) |
| | neg | neg (2) |
| Lymphoblastic (4) | +++ | +++ (1) |
| | +++ | ++ (1) |
| | +++ | + (1) |
| | +++ | neg (1) |

[a]Number of cases in each category; +++ = >70% of the cells staining; ++ 30–70%; + 5–30%; neg < 5% cells staining.

Cases were classified histologically using the Kiel Classification. Immunoperoxidase staining and scoring of frozen sections of tumour tissue was carried out as previously described (Krajewski *et al.*, 1985).

All cases were defined as B cell lymphoma by demonstrating staining with B-lineage specific monoclonal antibodies (Dako B or Coulter B1) and/or by demonstrating staining for immunoglobulin with light chain restriction.

Cases of lymphoblastic lymphoma all showed strong staining with J5 (CALLA) and with Dako B, but only one case stained for immunoglobulin (Mλ).

Cells were stained for total class II-positive cells with DA6.231 (DP+DQ+DR) and with TU22 and Leu10 (DQ-specific). TU22 and Leu10 gave identical results in all cases.

**Table III** Immunohistochemical staining of B-cell non Hodgkin's lymphoma with a panel of anti-class II monoclonal antibodies

| *Histopathological classification* | *Cells stained for: DP+DQ+DR (DA6.231)* | *DP (B7/21)* | *DR (L243)* | *DR (DA6.164)* | *DR+DQ (DA6.147)* | *DQ (Leu10)* | *DQ (TU22)* |
|---|---|---|---|---|---|---|---|
| Lymphocytic | +++ | +++ | ++ | ++ | ++ | ++ | ++ |
| Lymphocytic | +++ | +++ | +++ | +++ | ++ | ++ | ++ |
| Lymphocytic | +++ | +++ | +++ | +++ | +++ | neg | neg |
| Centrocytic (small cell) | +++ | +++ | +++ | +++ | +++ | neg | neg |
| Centrocytic (small cell) | +++ | ++ | +++ | +++ | ++ | + | + |
| Centrocytic (large cell) | +++ | +++ | nd | +++ | +++ | +++ | +++ |
| Hairy cell leukaemia | +++ | +++ | +++ | neg | +++ | neg | neg |
| Hairy cell leukaemia | +++ | +++ | +++ | +++ | +++ | +++ | +++ |
| Hairy cell leukaemia | +++ | +++ | +++ | +++ | +++ | +++ | +++ |
| CB/CC-follicular | +++ | ++ | + | + | + | +++ | +++ |
| CB/CC-follicular | +++ | ++ | nd | + | + | + | + |
| CB/CC-follicular | +++ | +++ | +++ | +++ | ++ | ++ | +++ |
| CB/CC-follicular | +++ | ++ | ++ | ++ | ++ | ++ | ++ |
| CB/CC-diffuse | +++ | +++ | nd | +++ | +++ | +++ | +++ |
| Centroblastic | +++ | +++ | + | neg | neg | neg | neg |
| Centroblastic | +++ | +++ | +++ | neg | +++ | neg | neg |
| Centroblastic | +++ | +++ | +++ | neg | neg | +++ | +++ |
| Immunoblastic | +++ | +++ | +++ | +++ | +++ | +++ | +++ |
| Immunoblastic | +++ | +++ | +++ | +++ | +++ | +++ | +++ |
| Immunoblastic | +++ | +++ | +++ | +++ | +++ | +++ | +++ |
| Immunoblastic | +++ | ++ | nd | ++ | ++ | ++ | +++ |
| Plasmacytoma | ++ | ++ | + | + | + | + | + |
| Plasmacytoma | neg | neg | neg | neg | neg | neg | neg |
| Plasmacytoma | neg | neg | neg | neg | neg | neg | neg |
| Lymphoblastic | +++ | +++ | +++ | +++ | +++ | ++ | ++ |
| Lymphoblastic | +++ | + | + | ++ | + | + | + |

CB/CC = centroblastic/centrocytic; nd = not done; neg = <5%; + = 5–30%; ++ = 30–70%; +++ = >70% cells staining.

examined (Shackelford *et al.*, 1982). This may suggest that products of the different DR beta chain genes are non-coordinately expressed. In all cases of NHL examined in the present study the anti-DQ antibodies TU22 and Leu10, which are directed to different determinants, have given equivalent results.

### Expression of MHC class II antigens in hairy cell leukaemia

Hairy cell leukaemia (HCL) is a rare variant of lymphoid malignancy, in which the cells are commonly of the B cell lineage (Hermann *et al.*, 1985). Faille and colleagues (1984) examined a single case of HCL by biochemical means and showed that the cells expressed DR antigens but failed to express DQ antigens. DQ expression was induced by treating the cells with TPA, sodium butyrate or 5-azacytidine. We have examined five subjects with hairy cell leukaemia and in all cases, DR antigens are detectable on the majority of the cells. In one case, sections of spleen have been examined by immunohistochemistry and DQ antigen expression is undetectable (Krajewski *et al.*, 1985). Two further cases examined by immunohistochemistry show coordinate expression of class II antigens (Table III). In two other cases, in which peripheral blood cells were examined by indirect immunofluoresence, DQ and DP antigen expression are detectable (in one case on almost all of the cells) (Figure 2). Cells have also been examined after culture with phorbol ester (*vide infra*).

In agreement with other studies (Korsmeyer *et al.*, 1983) we find readily detectable expression of interleukin-2 receptor on HCL cells in tissue sections and on peripheral blood cells in all cases.

### Expression of MHC class II antigens in common acute lymphoblastic leukaemia and in pre-B cell lines

Immunoglobulin gene rearrangements, and expression of cytoplasmic $\mu$ chains (after stimulation of ALL cells with phorbol ester) suggest that the

**Figure 2** Cells from two cases of hairy cell leukaemia before and after culture of the cells with TPA. Cells were analysed as described in **Figure 1**. In the first case (a), DP and DQ antigens are expressed on only a proportion of the cells before culture (i–v) and class II expression is much increased after culture with TPA (vi–x). In the second case (b), DP and DQ antigens are expressed on almost all of the cells before culture with TPA (i–v). After culture with TPA (vi–x) class II expression is much reduced and this involves reduction of DP, DQ and DR expression. DQ and DP expression are much reduced and detectable on only a very small proportion of the cells. Interleukin 2 receptor (anti-Tac antibody) is detectable in both cases on the untreated cells and is reduced in expression in both cases after culture of the cells with TPA.

cells in J5-positive, common acute lymphoblastic leukaemia (ALL) are committed to differentiation along a B cell pathway (Cossman *et al.*, 1982). There are apparently conflicting results on the expression of class II antigens in ALL, although there is no doubt that except in very rare cases, class II antigens are expressed: Newman & Greaves (1982) showed that of 556 cases of ALL examined only 7 failed to express detectable levels of class II antigens. We have examined only a small number of ALL (van Heyningen *et al.*, 1982; Guy *et al.*, 1986*a*). Nevertheless, it is obvious that heterogeneity of class II expression is as much a feature of ALL as it is of low grade leukaemia and other non-Hodgkin's lymphoma. We and others find that some ALL fail to express detectable levels of DP and DQ antigens, although the same cells are uniformly DR-positive (Newman *et al.*, 1983; Guy

*et al.*, 1986*a*). Other studies suggest that in some cases of ALL there are more cells with detectable expression of DP antigens than DR antigens (Wernet *et al.*, 1984) and that DQ expression is confined to a minority of the DR-positive cells.

The finding of heterogeneity of cell surface class II antigens in ALL is supported by quantitative studies in 37 cases of ALL, where it was found that the range of expression varied from patient to patient by more than ten fold (Okamura *et al.*, 1984). Similar results have been reported in CLL (Okamura *et al.*, 1982*a*).

Non-coordinate expression of DR and DQ antigens is also a feature of a number of established lines derived from cases of ALL (Ziegler *et al.*, 1982; Newman *et al.*, 1983; Wang *et al.*, 1983). That is, the majority of cells from the lines are DR-positive and fewer cells are DQ-positive. In the study of Wang *et al.* (1983) the monoclonal anti-DP antibody ILR-1 was also reported to react with the majority of cells in the lines.

### MHC class II determinants as allelic markers of B cell malignant disease

In a series of Edinburgh CLL patients studied, no evidence was found that MHC class I or class II antigens are markers of genetic susceptibility to B cell malignant disease. There was an association for a subgroup of patients lacking HLA-A1 and B8 with less need for treatment within a few months of diagnosis (Kilpatrick *et al.*, 1984). Other studies have described an association between expression of the DQ determinant recognized by the monoclonal antibody IVD12 (anti-DQw3) and CLL: in the study of Nunez–Roldan *et al.* (1982), 93% of patients with CLL were IVD12-positive (and DR4 or DR5-positive) in comparison with 50% of controls. Linkage disequilibrium accounts for the association of DQw3 with DR4 and DR5. In the Edinburgh study, only ~40% of the patients, and an equivalent number of controls, were DR4 and/or DR5 positive (D.C. Kilpatrick, personal communication). The reasons for the discrepancy may be a reflection of differences in the selection of patients, classification of the disease or of the ethnic origins of the subjects studied.

### Stimulation of increased levels of MHC class II antigen expression in CLL with phorbol ester

The croton seed oil tumour-promoting agent 12-O-tetradecanoyl phorbol-13-acetate (TPA), introduced several years ago (Totterman *et al.*, 1980), has been used widely in studies on malignant B cells. TPA is an extremely powerful tool for dissecting events contributing to differentiation of leukaemic cells. The effects of TPA on morphology of CLL cells and on their ability to secrete immunoglobulin are consistent with induction of differentiation of the cells (Totterman *et al.*, 1980; Okamura *et al.*, 1982*b*). Cells from some patients, but not others, enter S phase of the cell cycle after several days of culture with TPA (Godal *et al.*, 1982). In some cases there is also secretion of considerable quantities of immunoglobulin (reviewed by Gordon, 1984). In all cases, irrespective of whether the cells enter S phase and/or secrete immunoglobulin, class II expression is markedly increased by culture of the cells with TPA (Totterman *et al.*, 1981; Okamura *et al.*, 1982; Guy *et al.*, 1983*a*, 1983*b*; Guy *et al.*, 1986*a,b*). The increases in class II expression are highly variable from patient to patient but appear to involve increased expression of DP, DQ and DR antigens in all cases (Guy *et al.*, 1983*a*; Guy *et al.*, 1986*a, b*). An example of CLL cells examined before and after culture with TPA for 72 h is shown in Figure 3. In the example illustrated, class II expression is increased about five fold by culture with TPA. In other cases, where class II expression is especially low on the untreated cells, increments of more than twenty fold in DQ expression are found (Guy *et al.*, 1986*a*). In all cases studied, DQ and DP antigens are detectable on >85% of the cells after culture with TPA, even in those cases where DP and DQ expression is confined to about 20% of the cells before culture (Guy *et al.*, 1983*a*; Guy *et al.*, 1986*a*). Transferrin receptor and 4F2 expression are also increased by TPA treatment of the cells but the expression of Leu-1 is not substantially affected.

The appearance of increased levels of class II antigens at the cell surface in CLL is a reflection of increases in the steady state levels of class II specific mRNA transcripts, detectable with complementary DNA probes specific for DP, DQ and DR genes (Guy *et al.*, 1986*b*; and unpublished results). In samples examined from three patients, the levels of class II specific mRNA transcripts detected with cDNA probes parallel the levels of class II antigens at the cell surface detected with monoclonal antibodies. There is a need to establish in more extensive studies if the serological differences in class II expression at the cell surface are reflected at the mRNA level.

The proliferation centres of lymph node and involved splenic white pulp areas in B cell lymphocytic lymphoma preferentially stain with anti-DQ antibodies, in comparison with the surrounding cells, and as indicated previously (Swerdlow *et al.*, 1984) in this respect they are

**Figure 3** Analysis of cells from a second CLL patient (WT) illustrates the higher level of class II expression on cells from some patients (cf. **Figure** 1) and the increase in class II expression inducible with TPA. Cells from WT have been examined on a number of occasions and reported elsewhere (Guy *et al.*, 1986*a*). Cells were analysed as in **Figure 1.** About 50–60% of the cells are DP- and DQ-positive before culture with TPA (panels i–v) and >90% after culture (vi–x). Expression of transferrin receptor (detected with BK19/9) is also induced by culture with TPA and Leu-1 (non-class II antigen CD5) expression is stable. The increase in expression of class II antigens on cells of WT has also been quantified by absorption: DQ expression is increased about five fold and this represents the minimum increase in DQ expression seen in the present series after TPA activation of CLL cells (Guy *et al.*, 1986*b*).

similar to TPA-treated CLL cells. Recently we have found that proliferation centres also express 4F2 and transferrin receptors (unpublished results).

It is difficult to determine how far towards the terminal stages of differentiation CLL cells progress after culture with TPA. A further problem lies in distinguishing differentiation from activation of the cells. Nevertheless, it is clear that cells from B cell CLL patients are not in an intractable state of maturation arrest. This implies that genes which are important in controlling differentiation of the cells are intact and have retained susceptibility at least *in vitro* to epigenetic regulation.

Cells from two cases of hairy cell leukaemia (HCL) have been tested after culture with TPA (Figure 2). In one case class II expression is increased and in the other class II expression is substantially reduced. This is unexpected, because class II expression after culture of CLL cells with TPA is almost invariably increased. These findings may suggest that some HCL (before culture with TPA) are analogous to TPA-activated CLL cells, and are close to terminal differentiation and loss of class II expression, which then occurs when the cells are cultured with TPA. Interleukin-2 (IL-2) receptor expression is also reduced in HCL after culture of the cells with TPA. The loss of IL-2 receptor is consistent with the suggestion that some HCL are close to terminal differentiation, as is the finding that plasma cell associated antigens are expressed in HCL (cited in Hermann *et al.*, 1985).

In only a single case of plasma cell leukaemia have the effects of TPA on cells at the terminal stages of B cell differentiation been tested (Guy *et al.*, 1986*b*). Class II expression is not induced in a class II-negative, class I-positive plasma cell leukaemia, suggesting that the ability to express the antigens is irrevocably lost at the plasma cell stage.

Cells from some patients with B cell leukaemia respond to TPA stimulation with high rates of immunoglobulin secretion. This will provide a valuable source of material for the production of anti-idiotype monoclonal antibodies for possible therapeutic use.

### Functional consequences of anomalous MHC class II expression in B cell malignancy

A major mechanism which regulates the interactions of cells comprising the immune system, involves recognition of class II molecules through specific receptors on the surface of T lymphocytes (Biddison *et al.*, 1983). Variation in the levels of expression of class II antigens and differences in the expression of products of the different class II loci, are likely to influence cellular interactions in the immune system. Since class II antigens are also expressed on non-lymphoid tissues, including vascular endothelia (Gronewegen *et al.*, 1985; Muller *et al.*, 1985) these effects may be more widespread than is at first evident.

It is conceivable that some cellular interactions involving class II antigens might predispose to the activation of T lymphocytes with suppressor activity (Palacios *et al.*, 1983; Pawelec *et al.*, 1984). This would be a normal function of cellular immunity but could also be a contributory factor to the pathogenesis of malignancy of the B cell series. Among normal cells, both quantitative and qualitative differences in class II expression have functional consequences. For example, the ability of monocytes to present antigen to T cells correlates with the coordinate expression of DR and DQ antigens (Gonwa *et al.*, 1983). Class II-dependent allogeneic stimulation of T cells by monocytes is a function of quantitative variation in class II expression on the stimulator population (Nunez *et al.*, 1985). In this regard, cells from CLL patients are inefficient stimulators of allogeneic T lymphocytes (Halper *et al.*, 1979), unless the cells are first cultured with TPA (Okamura *et al.*, 1982*b*).

The growth and differentiation of B cells involves secretion of specific factors by T lymphocytes (reviewed by Howard & Paul, 1983) which bind to, and induce the maturation of the cells. Activation of B cells, and their entry into the cell cycle from a resting state, involves substantial increases in class II expression in both normal (Kehrl *et al.*, 1985) and, as shown here, in malignant populations. Is increased cell surface class II expression on TPA-treated CLL cells a stimulus for T cell activation and subsequent synthesis of B cell growth and differentiation factors? Some findings suggest that this may be so. The TPA-induced differentiation of CLL cells which is characterized by morphological change, immunoglobulin secretion and cell cycle S phase entry (Gordon, 1984) is dependent on autologous helper T lymphocytes (Danersund *et al.*, 1985). TPA-induced increases in class II expression are T cell independent (unpublished results). In preliminary experiments we find that S phase entry of CLL cells, which occurs after several days of culture with TPA, is blocked by some anti-class II monoclonal antibodies. This suggests that interactions of class II antigens on TPA-treated CLL cells with T cells may lead to activation of the T cells, and perhaps induction of B cell growth and differentiation factor synthesis. Therefore it is possible that deficiencies of expression of class II antigens in B cell malignancy contribute primarily to the pathogenesis of malignant disease, because of failure of the malignant cells to activate T lymphocytes *in vivo*. However, this requires much further analysis. An obvious difficulty in proposing deficiencies of class II expression as a mechanism contributing to the maturation arrest of malignant B cells, is that many cases of CLL and NHL have coordinate expression of class II antigens and very substantial quantities of the antigens at the cell surface. In the absence of additional anomalies affecting the ability of the cells to respond to differentiation signals, this would appear to be incompatible with a possible failure to activate T cells.

It is possible that determining the class II phenotypes of cells from leukaemia and lymphoma patients may be of some prognostic significance. It will be necessary to evaluate this by monitoring the survival of a number of patients with different patterns of class II expression over a long period of time. However, in the short term there does not appear to be any significant diagnostic or prognostic value in determining class II phenotypes, except perhaps in aiding the histopathological classification of non-Hodgkin's lymphoma. The class II phenotypes of some CLL patients are very stable with time (Guy *et al.*, 1986*a*) and are apparently unaffected by treatment of the disease.

The results of analysis of class II expression in CLL (and also in some normal tissues) imply that there may be independent regulatory elements for the different class II genes. This is in contrast to other studies which imply that the regulation of expression of products of the different class II genes is coordinate (Mach *et al.*, 1984). Furthermore, the expression of DQ antigens does not occur in malignant B cells unless there is also DR expression. This may suggest that there are regulatory mechanisms for DQ antigens which are inherent to the DR genes. The ability of CLL cells to respond with much increased class II expression after stimulation with TPA, will be of use in detailed molecular studies and the availability of large numbers of B cells from CLL patients will greatly help examination of the mechanisms of class II antigen regulation.

Further studies of cells from patients with B cell leukaemia and lymphoma will provide much valuable information on the structure and function of class II antigens, and this will be relevant to an understanding of the mechanisms governing their expression in normal tissues. The possible role of class II antigens in stimulating autologous cellular interactions, leading to the synthesis of growth and differentiation factors for normal B cells is crucial also to the understanding of failures of malignant B cell differentiation. With this knowledge the significance of anomalous expression of MHC class II antigens in the pathogenesis of B cell malignant disease may be revealed.

We are grateful to many colleagues who have very generously given reagents for use in these studies. Our thanks go to Dr C.M. Steel for his reading of this manuscript and to Dr G. Stockdill, without whose generous help in providing access to clinical material these studies would not have been possible. We thank Linda Docherty for valuable technical assistance and the Photography Section of MRC CAPCU for illustrations. AED and ASK gratefully acknowledge financial support from the Melville Trust.

**References**

ADDIS, J.B.L., TISCH, R., FALK, J.A. & LETARTE, M. (1982). The analysis with monoclonal antibodies of the heterogeneity of Ia glycoproteins on chronic lymphocytic leukaemia cells. *J. Immunol.*, **129**, 2033.

BIDDISON, W.E., RAO, P.E., TALLE, M.A., GOLDSTEIN, G. & SHAW, S. (1983). Possible involvement of the T4 molecule in T-cell recognition of class II antigens: Evidence from studies of proliferative responses to SB antigens. *J. Immunol.*, **131**, 152.

BODMER, J.G. & BODMER, W.F. (1984). Monoclonal antibodies to HLA determinants. *Brit. Med. Bull.*, **40**, 267.

BRODSKY, F.M. (1984). A matrix approach to human histocompatibility antigens: Reactions of four monoclonal antibodies with the products of nine haplotypes. *Immunogenetics*, **19**, 179.

BROWN, G., WALKER, L., LING, N.R. & 4 others. (1984). T-cell proliferation and expression of MHC class II antigens. *Scand. J. Immunol.*, **19**, 373.

CORTE, G., CALABI, F., DAMIANI, G., BARGELLESI, A., TOSI, R. & SORRENTINO, R. (1981). Human Ia molecules carrying DC1 determinants differ in both α- and β-subunits from Ia molecules carrying DR determinants. *Nature*, **292**, 357.

COSSMAN, J., NECKERS, L.M., ARNOLD, A. & KORSMEYER, S.J. (1982). Induction of differentiation in a case of common acute lymphoblastic leukaemia. *New Eng. J. Med.*, **307**, 1251.

CRUMPTON, M.J., BODMER, J.G., BODMER, W.F., HEYES, J.M., LINDSAY, J. & RUDD, C.E. (1984). In: *Histocompatibility Testing 1984*, Albert, E.D., Baur, M.P. & Mayr, W.R. (eds) pp. 29–37. Biochemistry of class II antigens: Workshop report. Springer-Verlag: Berlin. 1984.

DAAR, A.S., FUGGLE, S.V., FABRE, J.W., TING, A. & MORRIS, P.J. (1984). The detailed distribution of MHC class II antigens in normal human organs. *Transplantion*, **38**, 293.

DANERSUND, A., TOTTERMAN, T.H. & NILSSON, K., EGLE-JANSSON, I., KABELITZ, D. & SJOBERG, O. (1985). Phorbol ester-induced differentiation of chronic B lymphocytic leukaemia cells – regulatory impact of autologous and allogenic accessory cells. *Clin. Exp. Immunol.*, **59**, 644.

EDWARDS, J.A., JONES, D.B., EVANS, P.R. & SMITH, J.L. (1985). Differential expression of HLA class II antigens on human fetal and adult lymphocytes and macrophages. *Immunology*, **55**, 489.

FAILLE, A., TURMEL, P. & CHARRON, D.J. (1984). Differential expression of HLA-DC/DS molecules in a patient with hairy cell leukemia: Restoration of HLA-DC/DS expression by 12-O-tetradecanoyl phorbol-13-acetate, 5 azacytidine, and sodium butyrate. *Blood*, **64**, 33.

GILES, R.C. & CAPRA, J.D. (1985). Structure, function, and genetics of human class II molecules. *Adv. Immunol.*, **37**, 1.

GILES, R.C., DEMARS, R., CHANG, C.C. & CAPRA, J.D. (1985). Allelic polymorphism and transassociation of molecules encoded by the HLA-DQ subregion. *Proc. Natl Acad. Sci. USA.*, **82**, 1776.

GODAL, T., HENRIKSEN, A., RUUD, E. & MICHAELSEN, T. (1982). Monoclonal human B lymphoma cells respond by DNA synthesis to anti-immunoglobulins in the presence of the tumour promoter TPA. *Scand. J. Immunol.*, **12**, 267.

GONWA, T., PICKER, L.J., RAFF, H.V., GOYERT, S.M., SILVER, J. & STOBO, J.D. (1983). Antigen presenting capabilities of human monocytes correlates with their expression of HLA-DS, an Ia determinant distinct from HLA-DR. *J. Immunol.*, **130**, 706.

GORDON, J. (1984). Molecular aspects of immunoglobulin expression by human B cells leukaemias and lymphomas. *Adv. Cancer Res.*, **41**, 71.

GOYERT, S.M. & SILVER, J. (1983). Further characterization of HLA-DS molecules: Implications for studies assessing the role of human Ia molecules in cell interactions and disease susceptibility. *Proc. Natl Acad. Sci. USA*, **80**, 5719.

GRONEWEGEN, G., BUURMAN, W.A. & VAN DER LINDEN, C.J. (1985). Lymphokine dependence of in vivo expression of MHC class II antigens by endothelium. *Nature*, **316**, 361.

GUSTAFSSON, K., EMMOTH, E., WIDMARK, E., BOHME, J., PETERSON, P.A. & RASK, L. (1984). Isolation of a cDNA clone coding for an SB β-chain. *Nature*, **309**, 76.

GUY, K., VAN HEYNINGEN, V., COHEN, B.B., DEANE, D.L. & STEEL, C.M. (1982). Differential expression and serologically distinct subpopulations of human Ia antigens detected with monoclonal antibodies to Ia alpha and beta chains. *Eur. J. Immunol.*, **12**, 942.

GUY, K., RITCHIE, A.W.S., VAN HEYNINGEN, V. & DEWAR, A.E. (1983*a*). Further serological characterization of the heterogeneity of expression of human MHC class II antigens: Chronic lymphocytic leukaemia cells and monocytes differentially express DR and DC antigens. *Disease Markers*, **1**, 249.

GUY, K., VAN HEYNINGEN, V., DEWAR, A.E. & STEEL, C.M. (1983*b*). Enhanced expression of human Ia antigens by chronic lymphocytic leukaemia cells following treatment with 12-O-tetradecanoyl phorbol-13-acetate. *Eur. J. Immunol.*, **13**, 156.

GUY, K., DOCHERTY, L. & DEWAR, A.E. (1986*a*). Deficient expression of MHC class II antigens in some cases of human B cell leukaemia. *Clin. Exp. Immunol.* (In press).

GUY, K., MEEHAN, R.R., DEWAR, A.E. & LARHAMMAR, D. (1986*b*). Expression of MHC class II antigens in B cell chronic lymphocytic leukaemia, and increased levels of class II antigens and DR-specific mRNA after stimulation with 12-O-tetradecanoyl phorbol-13-acetate. *Immunology*. (In press).

HALPER, J., FU, S.M., WANG, C.Y., WINCHESTER, R. & KUNKEL, H.G. (1978). Patterns of expression of human Ia-like antigens during the terminal stages of B cell development. *J. Immunol.*, **120**, 1480.

HALPER, J.P., FU, S.M., GOTTLEIB, A.B. & WINCHESTER, R.J. (1979). Poor mixed lymphocyte stimulatory capacity of leukaemic B cells of chronic lymphocytic leukaemia patients despite the presence of Ia antigens. *J. Clin. Invest.*, **64**, 1141.

HERMANN, F., DORKEN, B., LUDWIG, W.D. & SCHWARTING, R. (1985). A comparison of membrane marker phenotypes in hairy-cell leukemia and phorbol-ester induced B-CLL cells using monoclonal antibodies. *Leuk. Res.*, **9**, 529.

VAN HEYNINGEN, V., GUY, K., NEWMAN, R. & STEEL, C.M. (1982). Human MHC class II molecules as differentiation markers. *Immunogenetics*, **16**, 459.

HOWARD, M. & PAUL, W.E. (1983). Regulation of B cell growth and differentiation by soluble factors. *Adv. Immunol.*, **1**, 307.

HSU, S-M. & JAFFE, E.S. (1984). Phenotypic expression of B-lymphocytes. Identification with monoclonal antibodies in normal lymphoid tissues. *Amer. J. Pathol.*, **114**, 387.

KEHRL, J.H., MURAGUCHI, A. & FAUCI, A.S. (1985). The modulation of membrane Ia on human B lymphocytes. *Cell Immunol.*, **92**, 391.

KILPATRICK, D.C., DEWAR, A.E., STOCKDILL, G. & 5 others. (1984). Histocompatibility antigen frequencies in patients with chronic lymphocytic leukaemia: Possible identification of a subgroup with relatively benign disease. *Scand. J. Immunol.*, **33**, 391.

KORMAN, A.J., BOSS, J.M., SPIES, T., SORRENTINO, R., OKADA, K. & STROMINGER, J.L. (1985). Genetic complexity and expression of human class II histocompatibility antigens. *Immunol. Rev.*, **85**, 45.

KORSMEYER, S.J., GREENE, W.C., COSSMAN, J. & 9 others. (1983). Rearrangement and expression of immunoglobulin genes and expression of TAC antigen in hairy cell leukemia. *Proc. Natl Acad. Sci. USA*, **80**, 4522.

KRAJEWSKI, A.S., GUY, K., DEWAR, A.E. & COSSAR, D. (1985). Immunohistochemical analysis of human MHC class II antigens in B-cell non-Hodgkin's lymphomas. *J. Path.*, **145**, 185.

LAMPSON, L.A. & LEVY, R. (1980). Two populations of Ia-like molecules on a human B cell line. *J. Immunol.*, **125**, 293.

MACH, B., DEPREVAL, C. & GORSKI, J. (1984). Molecular genetics of human class II genes and the regulation of their expression. In: *Progress in immunodeficiency research and therapy*. Griscell, C. & Vossen, J. (eds) p. 11. Elsevier: Amsterdam.

MARTI, G.E., NADLER, L.M., CHUSED, T.M., TOSATO, G., BLAESE, R.M. & KINDT, T.J. (1985). Cellular distribution of human I-A-like (DS/DC) antigen on normal and neoplastic lymphoid cells. *Human Immunol.*, **12**, 23.

MULLER, C., ZIEGLER, A., MULLER, C. & 4 others. (1985). Divergent expression of HLA-DC/MB, -DR, and -SB region products on normal and pathological tissues as detected by monoclonal antibodies. *Immunobiol.*, **169**, 228.

NATALI, P.G., SEGATTO, O., FERRONE, S., TOSI, R. & CORTE, G. (1984). Differential tissue distribution and ontogeny of DC-1 and HLA-DR antigens. *Immunogenetics*, **19**, 109.

NEWMAN, R.A. & GREAVES, M.F. (1982). Characterization of HLA-DR antigens on leukaemic cells. *Clin. Exp. Immunol.*, **50**, 41.

NEWMAN, R.A., DELIA, D., GREAVES, M.F., NAVARETTE, C., FAINBOIM, L. & FESTENSTEIN, H. (1983). Differential expression of HLA-DR and DR linked determinants on human leukaemias and lymphoid cells. *Eur. J. Immunol.*, **13**, 172.

NUNEZ, G., GILES, R.C., BALL, E.J., HURLEY, C.K., CAPRA, J.D. & STASTNY, P. (1984). Expression of HLA-DR, MB, MT and SB antigens on human mononuclear cells: Identification of two phenotypically distinct monocyte populations. *J. Immunol.*, **133**, 1300.

NUNEZ, G., BALL, E.J., MYERS, L.K. & STASTNY, P. (1985). Allostimulating cells in man. Quantitative variation in the expression of HLA-DR and HLA-DQ molecules influences T-cell activation. *Immunogenetics*, **22**, 85.

NUNEZ-ROLDAN, A., SZER, I., TOGUCHI, T., CUTTNER, J. & WINCHESTER, R. (1982). Association of certain Ia allotypes with the occurrence of chronic lymphocytic leukaemia. *J. Exp. Med.*, **156**, 1872.

OKAMURA, J., GELFAND, E.W. & LETARTE, M. (1982*a*). Heterogeneity of the response of chronic lymphocytic leukaemia cells to phorbol ester. *Blood*, **60**, 1082.

OKAMURA, J., LETARTE, M., STEIN, L.D., SIGAL, N.H. & GELFAND, E.W. (1982*b*). Modulation of chronic lymphocytic leukaemia cells by phorbol ester: Increase in Ia expression, IgM secretion and MLR stimulatory capacity. *J. Immunol.*, **128**, 2276.

OKAMURA, J., LETARTE, M. & GELFAND, E.W. (1984). Heterogeneity of non-T, non-B acute lymphoblastic leukemia defined by the quantitative expression of Ia and common ALL antigens. *Leuk. Res.*, **8**, 335.

PALACIOS, R., GUY, K. & VAN HEYNINGEN, V. (1983). Monoclonal antibodies against HLA-DR antigens acting on stimulator cells prevent OKT8+T lymphocytes from acquiring sensitivity to interleukin 2 and expressing suppressor activity. *Eur. J. Immunol.*, **13**, 64.

PAWELEC, G.P., SHAW, S., ZIEGLER, A., MULLER, C. & WERNET, P. (1982). Differential inhibition of HLA-D or SB-directed secondary lymphoproliferative responses with monoclonal antibodies detecting human Ia-like determinants. *J. Immunol.*, **129**, 1070.

PAWELEC, G., WERNET, P., ROSENLUND, R., BLAUROCK, M. & SCHNEIDER, E.M. (1984). Strong lymphoproliferative suppressive function of PLT clones specific for SB-like antigens. *Human Immunol.*, **9**, 145.

SHACKELFORD, D.A., KAUFMAN, J.F., KORMAN, A.J. & STROMINGER, J.L. (1982). HLA-DR antigens: Structure, separation of subpopulations, gene cloning and function. *Immunol. Rev.*, **66**, 134.

SCHLOSSMAN, S.F., CHESS, L., HUMPHREYS, R.E. & STROMINGER, J.L. (1976). Distribution of Ia-like molecules on the surface of normal and leukemic human cells. *Proc. Natl Acad. Sci. USA*, **73**, 1288.

SHAW, A.R.E., CHAN, J.K.W., REID, S. & SEEHAFER, J. (1985). HLA-DR synthesis induction and expression in HLA-DR negative carcinoma cell lines of diverse origins by interferon-$\gamma$ but not interferon-$\beta$. *JNCI*, **74**, 1261.

SHAW, S. & DEMARS, R. (1984). Binding specificity of anti-human Ia monoclonal antibodies analysed with HLA-deletion mutant cell lines: Patterns of binding to products of two different HLA haplotypes and of three subregions of a single haplotype. *Disease Markers*, **2**, 183.

SHAW, S., ZIEGLER, A. & DEMARS, R. (1985). Specificity of monoclonal antibodies directed against human and murine class II histocompatibility antigens as analysed by binding to HLA-deletion mutant cell lines. *Human Immunol.*, **12**, 191.

SRIGLEY, J., BARLOGIE, B., BULTER, J.J. & 7 others. (1985). Heterogeneity of non-Hodgkin's lymphoma probed by nucleic acid cytometry. *Blood*, **65**, 1090.

SWERDLOW, S.H., MURRAY, L.J., HABESHAW, J.A. & STANSFELD, A.G. (1984). Lymphocytic lymphoma/B-chronic lymphocytic leukaemia – an immunohisto-pathological study of peripheral B lymphocyte neoplasia. *Br. J. Cancer*, **50**, 587.

TOTTERMAN, T.H., NILSSON, K. & SUNDSTROM, C. (1980). Phorbol ester-induced differentiation of chronic lymphocytic leukaemia cells. *Nature*, **288**, 176.

TOTTERMAN, T.H., NILSSON, K., CLAESSON, L., SIMONSEN, B. & AMAN, P. (1981). Differentiation of chronic lymphocytic leukaemia cells in vitro. I. Phorbol ester-induced changes in the synthesis of immunoglobulin and HLA-DR. *Human Lymph. Diff.*, **1**, 13.

TROWSDALE, J. & KELLY, A. (1985). The human HLA class II $\alpha$ chain gene DZ$\alpha$ is distinct from genes in the DP, DQ and DR subregions. *EMBO J.*, **4**, 2231.

TROWSDALE, J., YOUNG, J.A.T., KELLY, A.P. & 8 others. (1985). Structure, sequence and polymorphism in the HLA-D region. *Immunol. Rev.*, **85**, 5.

WANG, C.I., AL-KATIB, A., LANE, C.L., KOZINER, B. & FU, S.M. (1983). Induction of HLA-DC/DS (Leu 10) antigen expression by human precursor cell lines. *J. Exp. Med.*, **158**, 1757.

WATSON, A.J., DEMARS, R., TROWBRIDGE, I.S. & BACH, F.H. (1983). Detection of a novel human class II HLA antigen. *Nature*, **304**, 358.

WERNET, P., MULLER, C.P. & OSTENDORF, P. (1984). Reactivity of lymphocytic and myelocytic leukemia blasts with monoclonal antibodies specific for the different human class II molecules. *Disease Markers*, **2**, 499.

ZIEGLER, A., UCHANSKA-ZIEGLER, B., ZEUTHEN, J. & WERNET, P. (1982). HLA antigen expression at the single cell level on a K562 X B cell hybrid: An analysis with monoclonal antibodies using bacterial binding assays. *Somat. Cell Genet.*, **8**, 775.